# Pulsed electromagnetic fields increase osteogenetic commitment of MSCs via the mTOR pathway in TNF-α mediated inflammatory conditions: an in-vitro study

**DOI:** 10.1038/s41598-018-23499-9

**Published:** 2018-03-23

**Authors:** Letizia Ferroni, Chiara Gardin, Oleg Dolkart, Moshe Salai, Shlomo Barak, Adriano Piattelli, Hadar Amir-Barak, Barbara Zavan

**Affiliations:** 1 Department of Biomedical Sciences, University of Padova, Via G. Colombo 3, 35100 Padova, Italy; 2 Division of Orthopaedic Surgery, Tel Aviv Sourasky Medical Center, Tel Aviv University Sackler Faculty of Medicine, Tel Aviv, Israel; 3 Private Practice, Tel Aviv, Israel; 4 Department of Medical, Oral, and Biotechnological Sciences, University of Chieti-Pescara, Chieti, Italy; 5 Department of Internal Medicine E, Tel Aviv Sourasky Medical Center, Tel Aviv University Sackler Faculty of Medicine, Tel Aviv, Israel

## Abstract

Pulsed electromagnetic fields (PEMFs) have been considered a potential treatment modality for fracture healing, however, the mechanism of their action remains unclear. Mammalian target of rapamycin (mTOR) signaling may affect osteoblast proliferation and differentiation. This study aimed to assess the osteogenic differentiation of mesenchymal stem cells (MSCs) under PEMF stimulation and the potential involvement of mTOR signaling pathway in this process. PEMFs were generated by a novel miniaturized electromagnetic device. Potential changes in the expression of mTOR pathway components, including receptors, ligands and nuclear target genes, and their correlation with osteogenic markers and transcription factors were analyzed. Involvement of the mTOR pathway in osteogenesis was also studied in the presence of proinflammatory mediators. PEMF exposure increased cell proliferation and adhesion and the osteogenic commitment of MSCs even in inflammatory conditions. Osteogenic-related genes were over-expressed following PEMF treatment. Our results confirm that PEMFs contribute to activation of the mTOR pathway via upregulation of the proteins AKT, MAPP kinase, and RRAGA, suggesting that activation of the mTOR pathway is required for PEMF-stimulated osteogenic differentiation. Our findings provide insights into how PEMFs influence osteogenic differentiation in normal and inflammatory environments.

## Introduction

Pulsed electromagnetic fields (PEMFs) have long been known to accelerate fracture repair^1^. Exposure to PEMFs has been shown to affect cell proliferation and differentiation by influencing multiple metabolic pathways, depending upon lineage and maturation stage. In the osteoblast lineage, PEMFs contribute to bone formation induced by a demineralized bone matrix and stimulate fracture healing, probably through the action of progenitors that are already committed towards bone^2^. Data on the mechanism of action of PEMFs and the potential involvement of specific signal transduction pathways are, however, scarce. It has been reported that PEMFs increase the activity of certain kinases belonging to known intracellular signaling pathways, such as the protein kinase A (PKA) and the MAPK ERK1/2^3,4^, and that they modulate anti-inflammatory effects by increasing the quantity of the adenosine receptors A2A^5^. PEMFs stimulation also upregulates BMP2 expression in association with increased differentiation in mesenchymal stem cells (MSCs)^6,7^.

Dental implants and total joint replacements are surgical procedures that involve the implantation of permanent biomaterials. An increasing number of these procedures has been extended to younger and middle-age patients, making long-standing biocompatibility, robustness and functionality crucial requirements for these implants. Despite many recent advances, revision surgeries of the implants continue to be a major concern due to the tissue response induced by implanted biomaterials, as well as the potential for loosening and periprosthetic osteolysis which remain significant challenges^8^.

The basis of recent insights into osseointegration range from the pure bone healing that takes place around the implant to an immune-mediated foreign body reaction^9–11^. That reaction involves a sequence of events, including protein adsorption on the surface of the implant, activation of complement and the coagulation system, recruitment of monocyte/macrophages and MSCs, activation and differentiation of these cells into functional macrophages, osteoclasts, and osteoblasts, respectively, and the formation of biological attachments between implant and new bone^11^. The continued release of wear debris from the implants and the potential evolving infection during the lifespan of the implant might induce peri-implant inflammation, resulting in peri-implant osteolysis, aseptic loosening and subsequent implant failure necessitating further surgical intervention^12^.

Serine/threonine kinase mammalian target of rapamycin (mTOR) has been shown to play an important role in osteoclast differentiation. It is activated by macrophage colony-stimulating factor, and its inhibition leads to decreased osteoclastogenesis^13,14^. Furthermore, mTOR expression levels are higher at the earlier stages of osteoclastogenesis and decrease at the later stages of osteoclast formation^14^. mTOR exists in cells as part of two complexes: complex 1 (mTORC1) and complex 2 (mTORC2). mTORC1 is activated by amino acids, growth factors, oxygen, inflammation, and Wnt signaling^15^. mTORC1 is also a negative regulator of autophagy, a lysosomal degradation process responsible for the removal of long-lived proteins and damaged organelles^16,17^. It has also been confirmed that the mTOR signaling pathway was involved in the regulation of apoptosis and autophagy in MSCs, and that its inhibition is able to attenuate age-related changes in MSCs^18^.

This study aimed to assess the potential involvement of the mTOR signaling pathway in the osteogenic differentiation of MSCs, the cells naturally involved in bone repair processes, under stimulation with PEMFs. To this end, we analyzed potential changes in the expression of mTOR signaling pathway components, including receptors, ligands and nuclear target genes, and their correlation with osteogenic markers and transcription factors. PEMFs were generated using a miniaturized electromagnetic device (MED) (Magdent Ltd., Tel Aviv, Israel) that is used successfully to stimulate implant osseointegration in the clinical setting and *in vivo* to^19^. The involvement of mTOR pathway in osteogenesis was also studied in the presence of proinflammatory mediators.

## Results

### Proliferation

**Figure 1:**
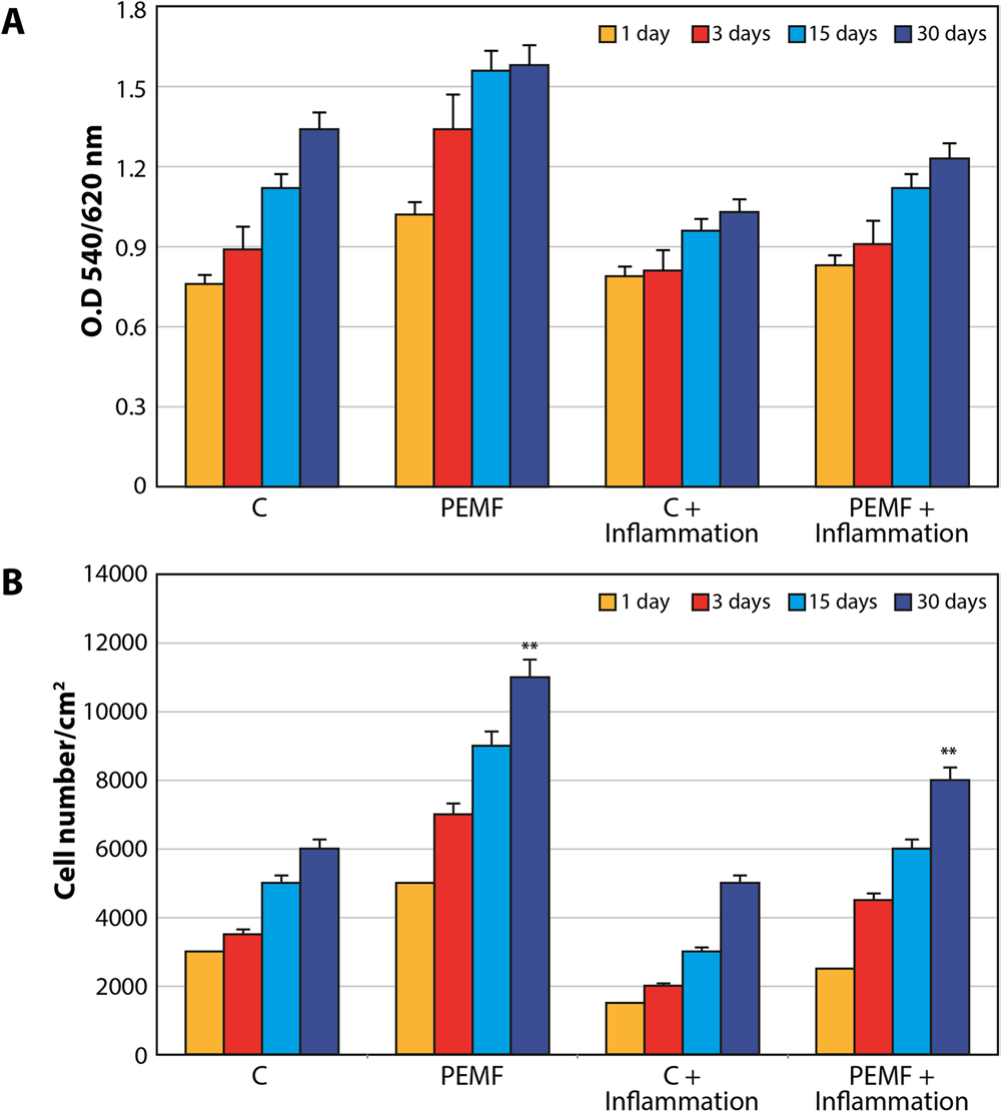
MSCs subjected to PEMF irradiation in the presence of proinflammatory cytokines for 30 days. (**A**) MTT proliferation assay. Results are expressed as mean ± SD of at least 3 independent experiments, *p < 0.05. (**B**) DNA content quantification. Results are expressed as mean ± SD of at least 3 independent experiments, *p < 0.05.

The biocompatibility of the surface was evaluated by MTT testing for measuring mitochondria activity as well as by evaluating cell numbers. Figure 1A displays the results of MTT testing conducted in normal conditions and in the presence of proinflammatory cytokines. Mitochondrial activity increased over time in both the control and PEMF groups. The presence of inflammatory cytokines caused a well-defined decrease in MTT values. The same pattern of increased cell proliferation was demonstrated by monitoring the cell numbers (Fig. 1B). Specifically, fewer cells were found in inflammatory conditions. Moreover, PEMF treatment was able to increase cell proliferation in both conditions. The proliferation rate was significantly higher in the PEMF group compared to the controls, even in an inflammatory environment.

### Morphology and cell adhesion properties

**Figure 2:**
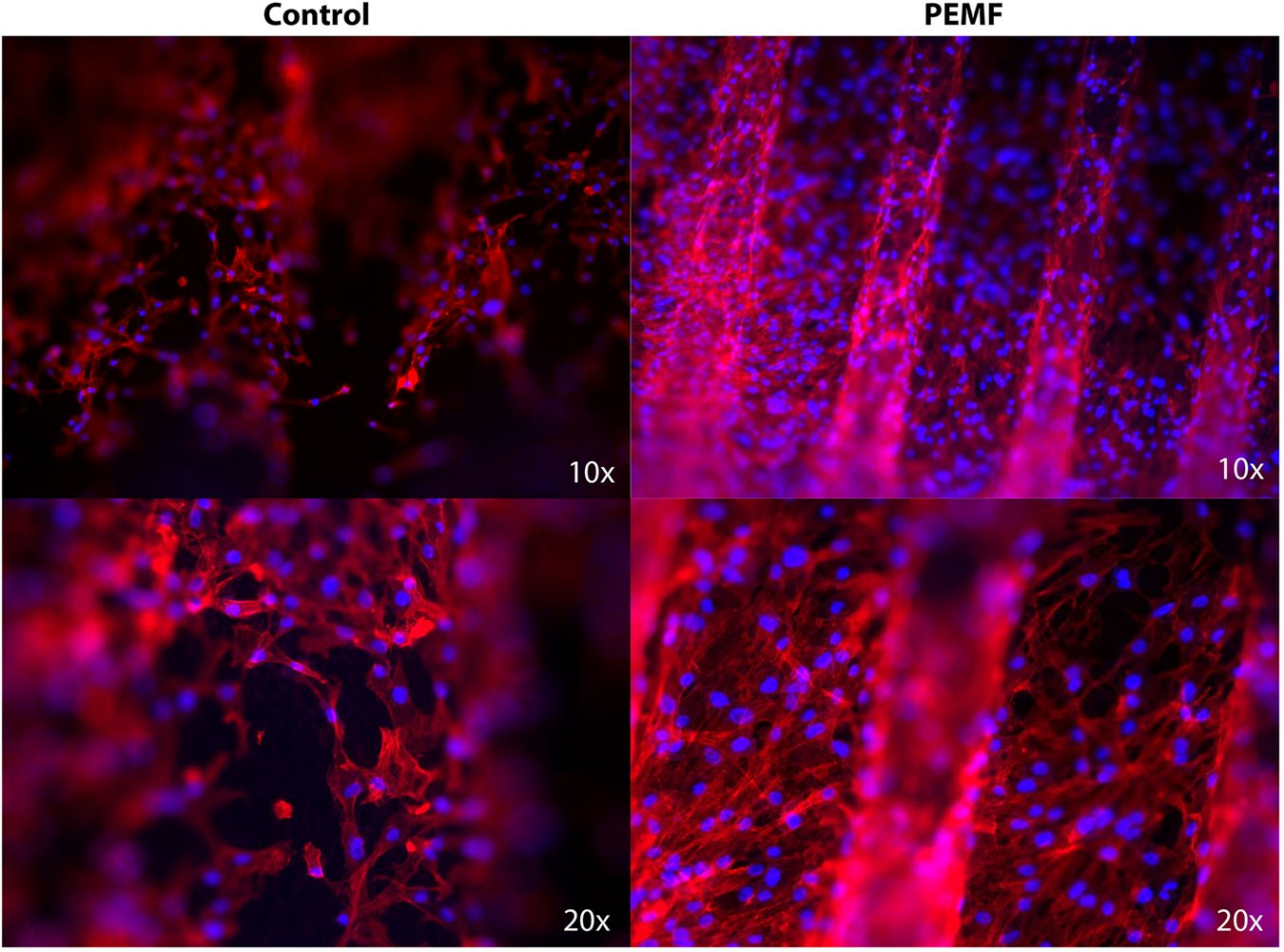
Morphologic analyses of MSCs. Phalloidin-labeled F-actin (red), DAPI nuclear staining (blue) and overlaid fluorescent image of immunostained cellular components (merged) for the MSCs of the control and PEMF-treated groups. After 7 days of culture, the cells were well-colonized throughout the implant surface, demonstrating a star-like shape associated with osteoblastic features. The cells were also able to spread after 7 days. PEMF irradiation resulted in a greater number of cells that were attached to the surfaces.

Morphologic analyses of MSCs were performed. Phalloidin-labeled F-actin (red), DAPI nuclear staining (blue) and overlaid fluorescent image of immunostained cellular components (merged) for the MSCs of the control and PEMF-treated groups are seen in Fig. 2. As shown in Fig. 2, the cells were able to attach to the implant surface in both the PEMF and control groups. The number of cells present on the implant surface with PEMF was clearly higher compared to the number of cells in the control group.

**Figure 3:**
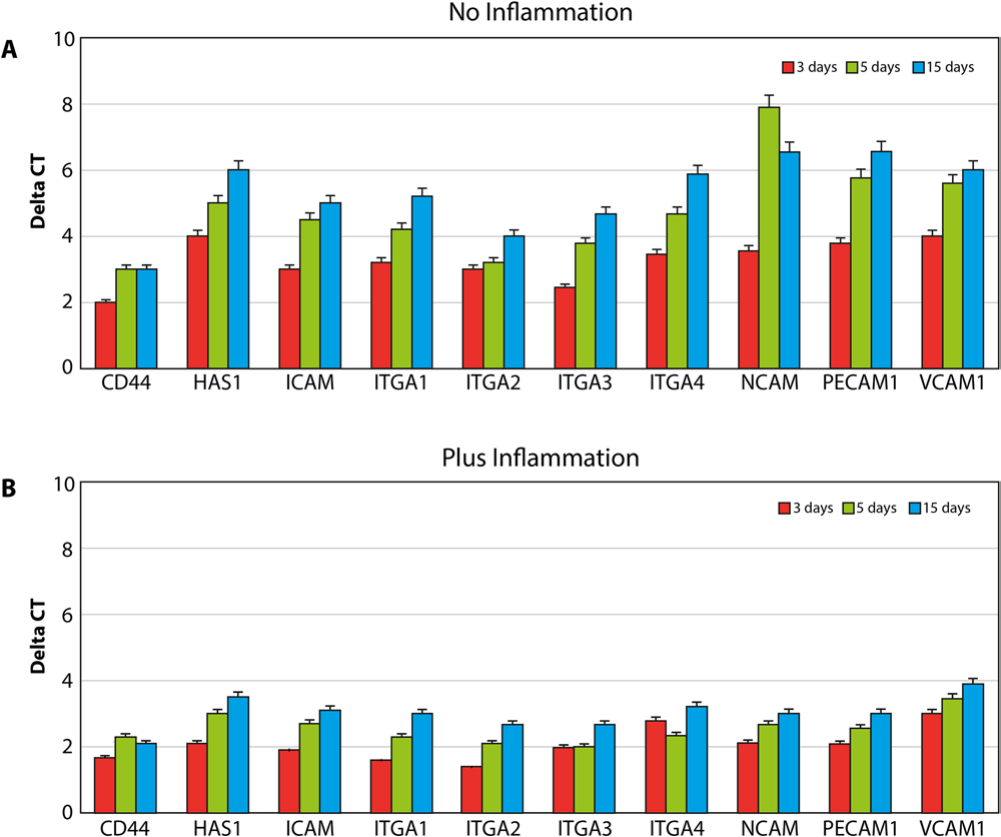
Analyses of cell adhesion properties in normal conditions (**A**) and in the presence of inflammation (**B**) were conducted by searching for the expression of molecules involved in hyaluronian synthesis (HAS1), i.e., extracellular receptor for hyaluronic acid (CD44), integrin (ITGA1, 2, 3, 4), and cadherin family cell adhesion molecules (NCAM; VCAM; PCAM). The results are reported as an increase in the gene expression value in samples of cells cultured on implants with MED device compared to the same gene expression obtained in normal conditions.

Cell adhesion properties were assessed by the analyses of gene expression of molecules involved on hyaluronan synthesis (HAS1), including receptor for extracellular hyaluronic acid molecules (CD44), integrin (ITGA1, 2, 3, 4), and cell adhesion molecules of the cadherine family, such as NCAM, VCAM, and PCAM (Fig. 3). The results are reported in all the graphs as an increase of gene expression value in samples of cells cultured in control conditions compared to cells exposed to PEMFs. PEMFs generated by MED were able to induce an increase in the expression of all these molecules, thereby confirming that they may enhance the adhesion properties of the cells. The presence of inflammatory stimuli (Fig. 3B) resulted in a reduction of cell adhesion, however, the presence of a PEMF significantly increased the expression of the integrin and cadherin receptors, thus potentially improving the ability of the cell to attach to the surface.

### Osteogenic process

**Figure 4:**
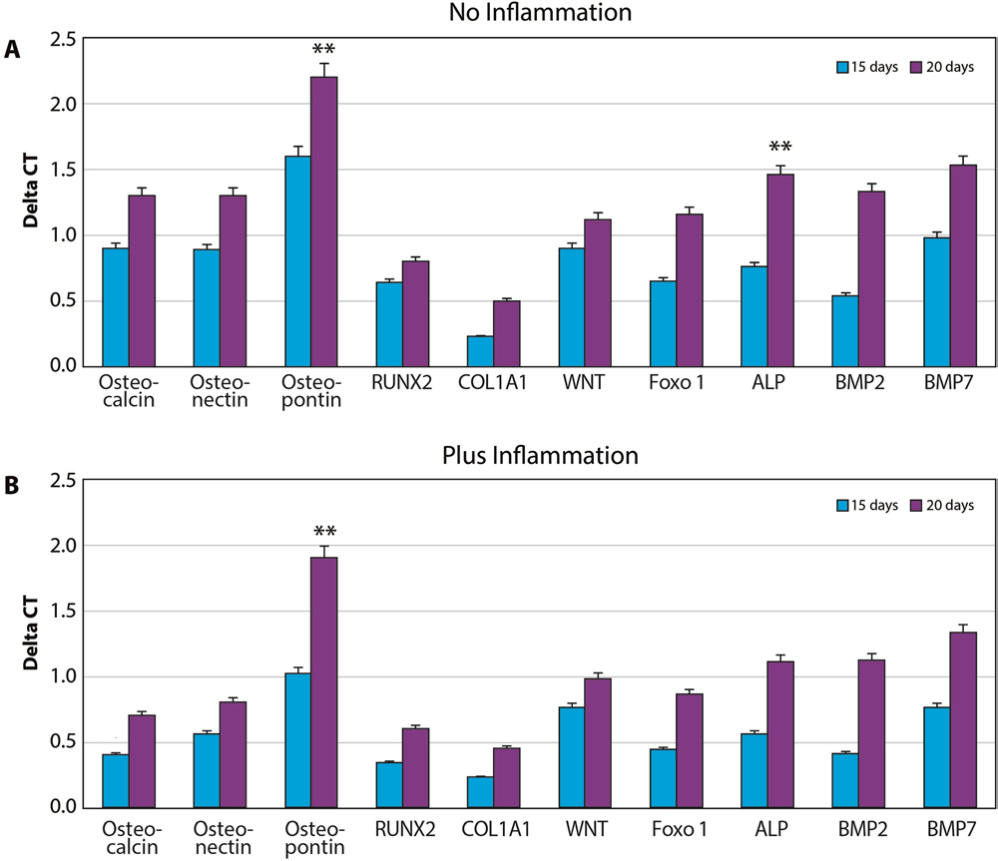
Real-time PCR for principal osteogenic markers, such as Runx, osteopontin, osteonectin, osteocalcin, collagen type I, wnt, foxO, ALP, BMP2, and BMP7 was performed in order to evaluate the commitment of stem cells onto an osteoblastic phenotype. The cells were cultured in the (**A**) presence and (**B**) absence of inflammatory conditions, and the variations obtained in normal implants versus implants + MED were compared.

**Figure 5:**
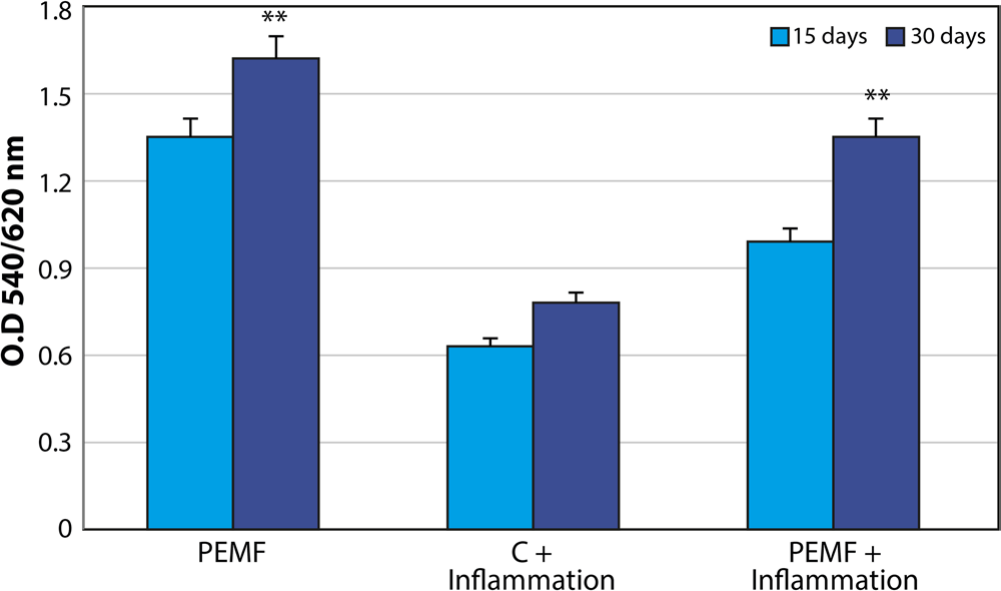
Quantification of intracellular ALP activity (expressed as U/mL) in MSC exposed to PEMFs and in non-exposed MSC in the presence and absence of an inflammatory environment at 15 and 30 days. Results are expressed as mean ± SD of at least 3 independent experiments, *p < 0.05. *; **p = 0.01; ***p = 0.001.

Real-time PCR for principal osteogenic markers, such as Runx, osteopontin, osteonectin, osteocalcin, collagen type I, wnt, foxO, ALP, BMP2, and BMP7 was performed in order to evaluate the commitment of MSCs onto osteoblastic phenotypes. The cells were cultured in the presence (Fig. 4A) and in the absence of inflammatory conditions (Fig. 4B) in order to compare the variations obtained in the control group with those obtained in the PEMF group. As illustrated in Fig. 4, in all the conditions an increase in expression of all osteogenic markers was noticed, confirming that the presence of PEMF exerts a positive effect on this process even in the presence of inflammatory cytokines. This commitment was confirmed by quantified ALP activity when MSCs were cultured in both the control and PEMF groups in the presence and absence of inflammatory stimuli (Fig. 5). Additionally, PEMFs were also able to induce a positive effect on the osteogenic process. It was clear that MSCs were also able to produce higher values of ALP in the presence of inflammatory cytokines as well. There was a significant, time-dependent ALP activity for cells grown under PEMF treatment, demonstrating the promotion of the crystallization of hydroxyapatites, a typical feature of pre-osteoblastic cells.

### mTOR pathway

In order to test if PEMF is able to excerpt its osteogenic properties thought mTOR pathway we used rapamicin to verify following hypothesis:

1. rapamicin is able to reduce the osteogenic properties in absence of PEMF(control);
2. The exposure to PEMF in presence of rapamicin could restore the osteogenic commitment of MSCs.

**Figure 6:**
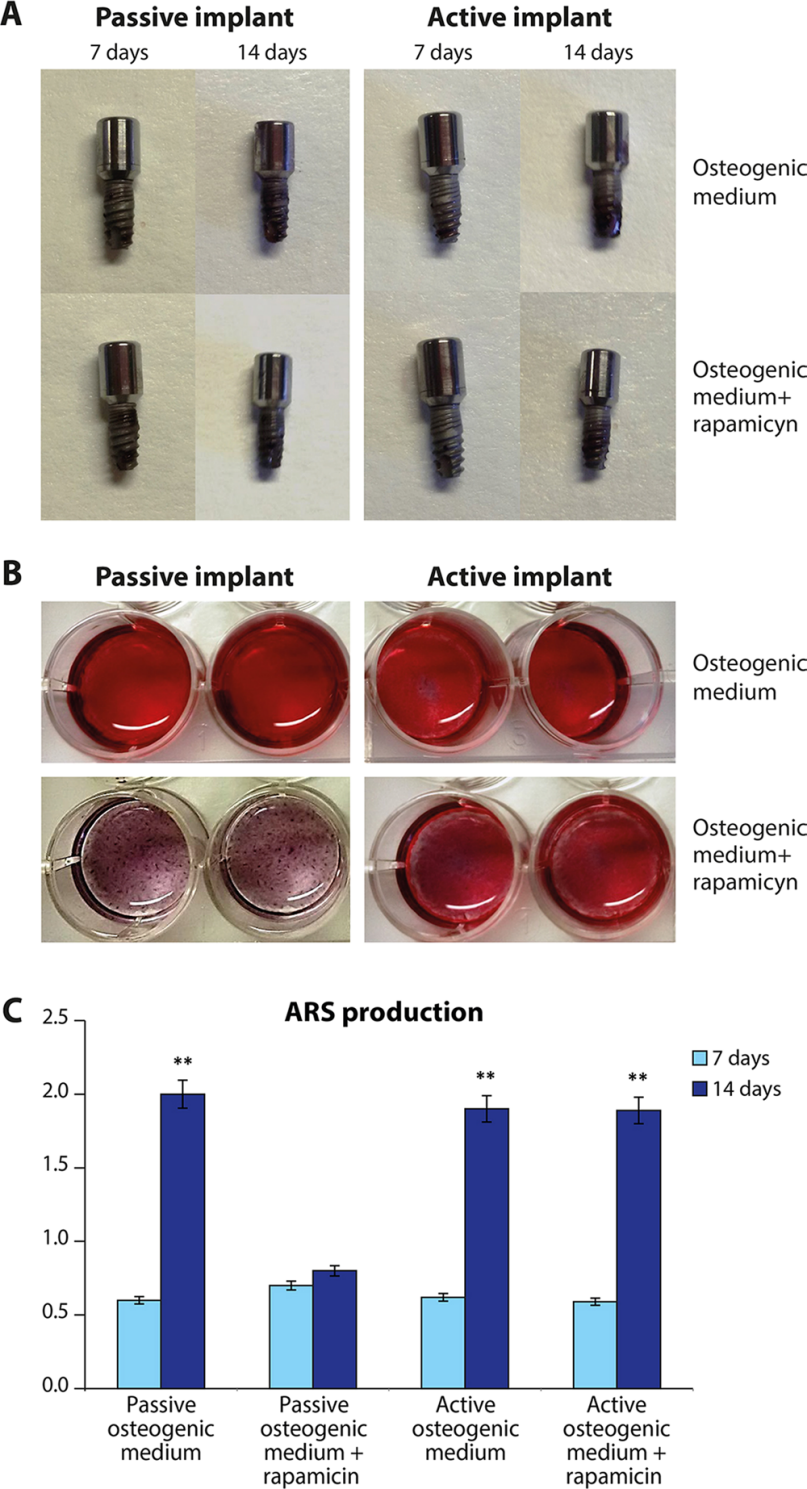
The osteogenic properties of MSCs seeded in the osteogenic medium have been evaluated as their ability to produce a mineralized extracellular matrix by means the ARS test. staining on implant (**A**); on the medium (**B**) and the quantification of ARS staining (**C**). Results are expressed as mean ± SD of at least 3 independent experiments, **p = 0.01.

The osteogenic properties of MSCs seeded in the osteogenic medium have been evaluated as their ability to produce a mineralized extracellular matrix by means the ARS test. Figure 6 reports the staining on implant (A); on the medium (B) and the quantification of ARS staining (C). The osteogenic potential is related to the ability to produce a mineralized matrix. Higher values of mineralization are represented by a greater values of the red staining (Fig. 6A,B). Spectroscopy was used to assess these parameters. The quantification of the osteogenic potential is reported in Fig. 6C. It is well evident that both in normal condition (passive implant) and in presence of PEMF (active implant) a decent quantity of ARS is detectable at the time frame of 7 to 14 days. When Rapamicin was added, a well-defined decline was noticed, predominantly at 14 days in passive condition. On the contrary, in presence of PEMF, Rapamicin was not able to inhibit the process and mineralization of the extracellular environment was demonstrated.

**Figure 7:**
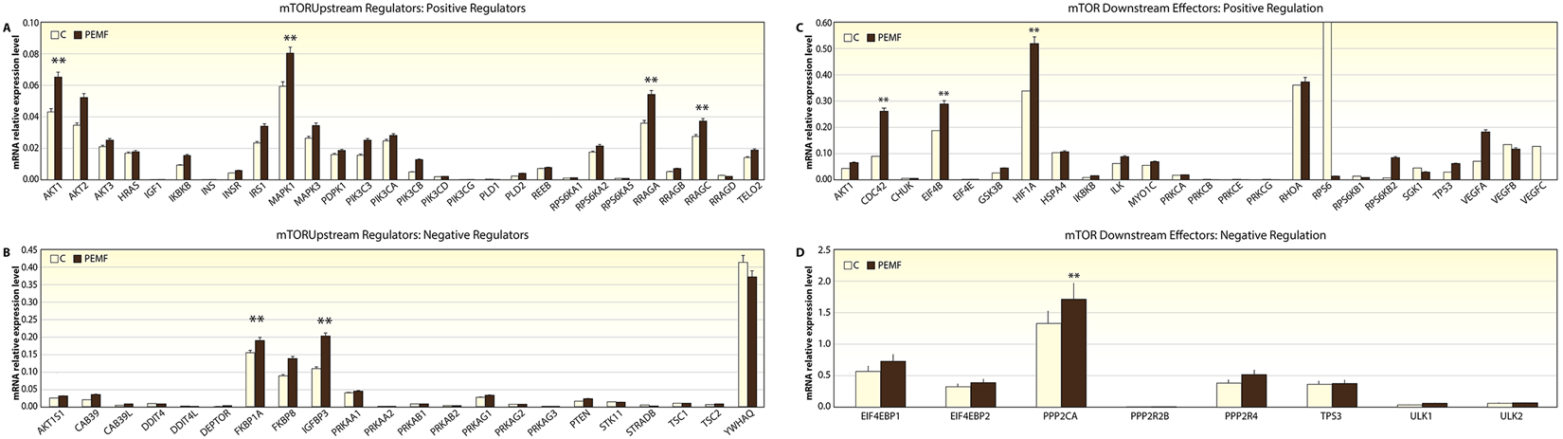
Gene expression of mTOR activity: (**A**) positive regulator, (**B**) negative regulator. (**C**) downstream effector: positive regulation, and (**D**) downstream effector: negative regulation.

**Figure 8:**
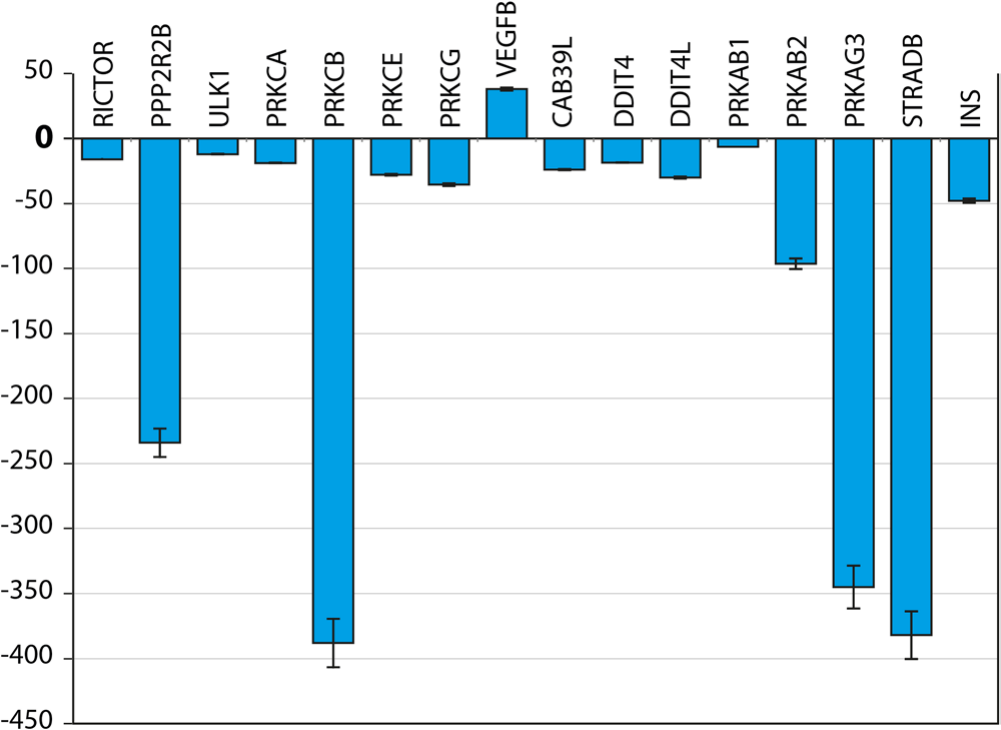
Real-time PCR analysis of mTOR pathway markers. Gene expression levels of the selected markers are reported as ration of MSC coltured on active implants in presence of osteogenic medium and Rapamicin implants with passive implants in presence of osteogenic medium and Rapamicin. Results are expressed as mean ± SD of at least 3 independent experiments, **p = 0.01.

Similar phenomenon was observed at gene expression level as well. In Figs 7 and 8 we report the gene expression of markers related to mTOR pathway evaluated at 14 day on MSCs cultures seeded in osteogenic medium with rapamicin, with or without PEMF treatment (passive VS active implant). Results have been grouped in correlation to their involvement in mTOR pathway: mTOR1 Complexes; mTOR2 Complexes; mTOR Upstream Regulators - negative regulation; mTOR Upstream Regulators - positive regulation; mTOR Downstream Regulators - negative regulation; mTOR Downstream Regulators - positive regulation. The results were analyzed and are presented as the ratio between: active implant in osteogenic medium + rapamicin with the active implant in osteogenic medium without rapamicin; passive implant in osteogenic medium + rapamicin with the passive implant in osteogenic medium without rapamicin. Value comprises from −2 to +2 are related to no significant variation. All genes related to the ratio in presence of a PEMF (active implant) are from −2 to +2, indicating that no difference occurs in co-presence of rapamicine and PEMF. On the contrary in absence of PEMF defined up or dowregulation to gene related to mTOR1 involved mostly in Adipogenic commitment rather than osteogenic commitment of MSCs were demonstrated.

In order to highlight genes responsive to the PEMF stimulus we analyzed also the results related to the ratio of gene expression of active implant in presence of osteogenic medium plus rapamicin with the passive implant in osteogenic medium + rapamicine (Fig. 7).

As reported in Fig. 8, a significant difference was found in presence of PEMF and is related to:

•Decrease in RICTOR (receptor for mTOR2)
•Decrease in Protein phosphatase 2, regulatory subunit B, beta (PPP2R2B) involved on mTOR2 pathway
•Decrease in PKC protein (involved on Adipogenesis)
•Increase on VEGF (involved on angiogenesis)
•Decrease in Upstream regulator of negative mTOR regulator:

Protein kinase, AMP-activated, beta 1 non-catalytic subunit (PRKAB1)

Protein kinase, AMP-activated, beta 2 non-catalytic subunit (PRKAB2)

Protein kinase, AMP-activated, gamma 3 non-catalytic subunit (PRKAG3)

•Decrease in downstream stream regulator of negative mTOR regulator:

Calcium binding protein 39-like (CAB39L)

DNA-damage-inducible transcript 4 (DDIT4)

DNA-damage-inducible transcript 4-like (DDIT4L)

STE20-related kinase adaptor beta (STRADB)

The results indicated that PEMFs enhance mTOR signaling by inducing an increase in the value of its related proteins, such as AKT, MAPP kinase, and RRAGA. Additionally, a significant increase in Rho family of GTPases was detected. Rho family members play crucial roles in mechanical signal transduction and promote the differentiation of MSCs into osteoblasts.

### Interleukin expression

**Figure 9:**
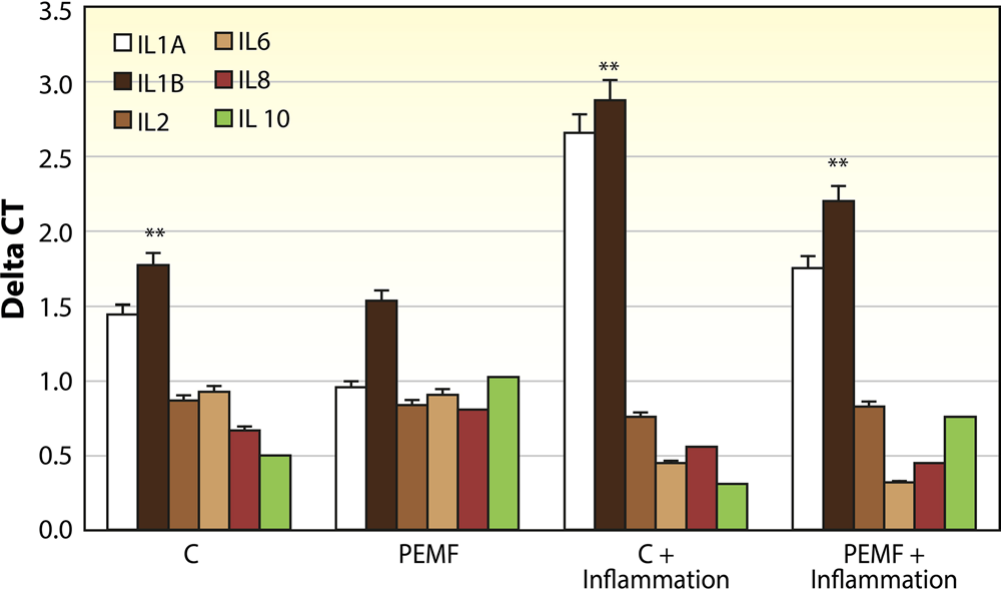
MSC were treated with inflammatory cytokines in the presence and absence of PEMFs. The results of the effect on inflammatory/anti-inflammatory activities of the active implants on MSC indicate a significant increase of *in vitro* expression of IL-10 (that exerts anti-inflammatory activity) in the presence of PEMFs generated by the MED device. Conversely, there is a reduction of expression of inflammatory cytokines, such as IL-1, in the presence of PEMFs. No significant difference in the expression of the other tested cytokines is evident.

MSCs were treated with PEMF in the presence of inflammatory cytokines as well as in the presence of PEMF. The results of their effect on inflammatory/anti-inflammatory activities of a PEMF are shown in Fig. 9, and they indicate that the presence of a PEMF induced a significant increase of *in vitro* expression of IL-10 (that exerts anti-inflammatory activity). Conversely, there was a reduction of expression of pro-inflammatory cytokines, such as IL-1, following PEMF treatment. There was no significant difference in expression of the other selected cytokines.

## Discussion

The principal results of the present study revealed several novel findings regarding the events involved in the stimulation of the osteogenic differentiation of MSCs induced by PEMFs. They identified a significant role of mTOR signaling during the differentiation driven by PEMF stimulation in an osteogenic microenvironment. Additionally, PEMFs were able to preserve the proliferation rate of MSCs in inflammatory conditions equal to that in a normal environment. MED-induced PEMF treatment resulted in an immunomodulatory effect in MSCs as expressed by increased IL-10 secretion. We found that PEMF stimulation of MSC proliferation mainly affected cell cycle regulation, cell structure, extracellular matrix, and some growth receptors involved in kinase pathways.

The osteointegration process begins with an inflammatory stage followed by the migration of MSCs. One of the major goals of dental, orthopedic and maxillofacial surgery is to achieve good and rapid osteointegration between implants and bone. The main research strategies to reduce implant failure aim at improving biomaterial characteristics, or stimulating bone endogenous repair, through a careful assessment of both processes by means of in *vitro* and in *vivo* experimental models before any application in humans. It had been reported that MED-generated PEMFs stimulated early bone formation around dental implants, already resulting in higher peri-implant bone-implant contact and bone mass after only 2 weeks, which suggests an acceleration of the osseointegration process by more than 3-fold^19^. However, the exact biologic mechanism of the influence of PEMFs on bone regeneration remains to be elucidated. A recent study by Ferroni *et al*. concluded that PEMFs affect the osteogenic differentiation of MSCs only if they are pre-committed, and that this therapy can be an appropriate candidate for the treatment of conditions requiring an acceleration of the repair process^20^.

We raised two major questions concerning the PEMF-related mechanism in the current study. First, we looked into the effects of PEMFs on MSCs in an inflammatory environment with regard to the ability of the cells to proliferate and adapt to the immunomodulatory changes. Understanding the mechanism of the implant’s integration, particularly the inflammatory response, is relevant for finding new treatment modalities to optimize the osteointegration and subsequent stability of the implants, which have implications in dentistry and orthopedic surgery. In this study, we added pro- and anti-inflammatory cytokines, which simulate the kinetics of their expression during early stages of implant integration *in vivo*, and investigated their effects on the proliferation and osteogenic differentiation of MSCs under PEMF irradiation. The proliferative capacity of MSCs is highly relevant for tissue repair^21^. Cytokines are known to affect proliferation of different cell types^21^. Therefore, we first analyzed the effect of selected cytokines on MSC proliferation. To the best of our knowledge, no previous study had assessed the influence of PEMF irradiation on the production of cytokines in MSC cultures. There are published data on the post-irradiation release of cytokines in mature osteoblasts^22^ and in osteoclast-like cells^23^. In both of those studies, ELISA was used for quantification and demonstrated an increase of TNF-a, IL-1b and PG-E2 in relation both to the recruitment of the osteoclast-like cells and to the intensity of the electrical field. The current study demonstrated the ability of MED-generated PEMFs to alter the immuno-modulative activity properties of MSCs. A significant elevation in anti-inflammatory cytokines, such as IL-10, was clearly present when MSCs were seeded on implants. IL-10 acted as an anti-inflammatory substance by inhibiting the synthesis of proinflammatory cytokines, and its up-regulation in MSCs may counteract the detrimental proinflammatory effects.

Second, we examined the effects of PEMFs on the mTOR signaling pathway, and the results confirmed that PEMFs in the presence of an inflammatory environment positively affected MSC commitment into an osteoblastic phenotype through the mTOR pathway. In *in vivo* model demonstrated that the IGF-1 released from the bone matrix during bone remodeling stimulated osteoblastic differentiation of recruited MSCs by activation of Akt/mTOR^24^. It had been reported that the presence of a good bone-like extracellular matrix was able to maintain bone mass by activation of mTOR in mesenchymal stem cells^24^. We now demonstrated that PEMF irradiation positively stimulated mTOR signaling, thus increasing the osteoblastic commitment of MSCs in the presence of inflammatory stimuli as well. This commitment could also be induced by increased integrin expression, such as α(4)β(1) integrin that has a high affinity for bone and improves the homing of MSCs to bone, thus promoting osteoblast differentiation and bone formation. mTOR is a central molecule in the regulation of cell growth in a wide variety of cells including osteoblasts, adipocytes, and myocytes. mTOR interacts with several proteins to form two distinct complexes named mTOR complex 1 (mTORC1) and 2 (mTORC2) which differ in their unique components, Raptor and Rictor. Upstream regulation and downstream products of mTORC1 are much more investigated than that of mTORC2. Though it is widely believed that the inhibition of mTOR signaling can promote osteoblastic differentiation, this issue is still controversial. While rapamycin primarily inhibits mTORC1, prolonged exposure can also disrupt mTORC2 function^25^. This fact makes difficult the data interpretation regarding the role played by mTORC1 and mTORC2 in osteogenesis. Martin SK *et al*. demonstrated that using Cre-mediated gene deletion in well established *in-vitro* differentiation assays, have shown that mTORC1 and mTORC2 have distinct roles in MSCs fate determination^26^. In agreement with previous studies^26,27^, blockade of Raptor in MSCs resultedin reduced adipogenic potential. Under osteoinductive conditions however, Raptor blockade promoted osteogenic differentiation. In current study we demonstrated that in osteogenic medium rapamicin is able to significantely reduce the mineralization of extracellular matrix. However, PEMF treatment is able to abolish this event, ensuring a good mineralization of extracellular environment. In light of these findings, we can assume that in presence of PEMF, the effect of rapamicin on osteoblasts behavior could be the opposit. Gene expression of 84 markers associated with mTOR pathway confirmed that no notable change in gene expression ocurred following rapamicin treatment coadministered with PEMF. While comparing gene expression under rapamicin treatment with PEMF to passive implant, reduction in mTOR2 pathway related genes was found. Namely, we found a reduction in Rictor expression that is associated to an adipogenic commitment of MSCs; and a decrease in several markers associated to a negative regulation of mTOR in both downstream and upstream levels. The most important changes are related to PKCβ that, as we have previously demonstrated is strongly related to the adipogenic commitment of MSCs^28–31^. We showed that PKCβ recruits the 66-kD proapoptotic isoform of Shc (p66Shc) to act as oxidoreductase within mitochondria and in triggering a feed-forward cycle of ROS production, eventually leading to cell death. The same players may come together in a radically different context, i.e., the production of cellular signals linking hyperglicemia to the regulation of a transdifferentiation scheme of stem cells residing in adipose tissues. Moreover a downregulation of genes related to adipofunction such as PRKAG3 involved on insulin signalling is well evident. Finally, a significant increase in VEGF gene was demonstrated. These data confirm the ability of PEMF to promote angiogenesis, that is cruicial during tissue regeneration as we have previously demonstrated in wound healing processes^32,33^.

The differentiations of MSCs into the osteoblastic or adipogenic lineages are inter-dependent process: molecular components promoting one cell fate inhibit the mechanisms leading the differentiation of the alternative lineage. Interestingly, inducers of differentiation along one lineage often inhibit differentiation along the other^34^. Our results suggest that in presence of osteogenic medium, PEMFs are able to induce osteogenic commitment of MSCs blocking the pathway of adipogenesis via mTOR related proteins.

This study reaults are in a line and comparable with a several previusly published papers. Ardeshirylajimi *et al*.^35^ investigated the the influence of prolonged pulsed extremely low frequency electromagnetic field on the osteogenic potential of cultured induced pluripotent stem cells. They concluded that combination of osteogenic medium and pulsed extremely low frequency electromagnetic field can be a great enhancement for bone differentiation of stem cells and appropriate candidate the management of bone defects and patients suffering from osteoporosis. A recently published paper by Arjmand *et al*.^36^ investigated the osteoinductive potential of PEMF in combination with Poly(caprolactone) (PCL) nanofibrous scaffold. Their results confirmed that the effects of PEMF on the osteogenic differentiation of ADSCs are very similar to these of osteogenic medium. They concluded that due to the immunological concerns regarding the application of bioactive molecules for tissue engineering, PEMF could be a good alternative for osteogenic medium. Additional recent article by Ardeshirylajimi *et al*.^37^ demonstrated that PEMF alone can induce osteogenic differentiation, but this capability was significantly increased when used in combination with electrospun polycaprolactone nanofibers. In addition, simultaneous use of osteogenic medium, PEMF and electrospun nanofibers resulted in increased osteogenic differentiation potential of induced pluripotent stem cells.

This study has several limitations, including its *in vitro* nature. Furthermore, the cells were grown in a monolayer, which does not accurately reflect *in vivo* conditions. The primary human cell cultures, however, can serve as a relevant model for examining the effects of PEMFs on bone cell physiology. The modulation of bone cell proliferation markers observed in this study have implications with regard to the immediate effects of PEMFs on bone formation and healing, as well as possible long-term implications for PEMF treatment.

In summary, the findings of the present study revealed that MED-generated PEMFs stimulate osteogenic differentiation and the maturation of the adipose tissue-derived MSCs via activation of the mTOR pathways. We also demonstrated that PEMF exposure increased cell proliferation, adhesion and the osteogenic commitment of MSCs, even in inflammatory conditions. We showed that PEMFs increased the expression of anti-inflammatory cytokines, such as IL-10, and reduced the expression of the pro-inflammatory cytokine IL-1. MSCs provided not only cell sources for connective tissues, but also had a significant influence on the immune response. Further studies are required to investigate the precise mechanisms by which mTOR signaling pathways are influenced and to discover other potential pathways involved in the PEMF-induced osteogenic effects.

## Methods

### PEMF exposure

The miniaturized electromagnetic device (MED) (Magdent Ltd., Tel Aviv, Israel) was the generator used to stimulate the cells. In the clinical setting, MED technology is used to actively stimulate osteogenesis and osseointegration. The MED was used with a Classix Dental Implant (3.3 mm 10 mm L Non Touch Prime, Cortex Ltd., Shlomi, Israel). The cells were irradiated continuously for 30 days with the MED inside the incubator and under the same conditions of temperature, humidity and CO_2_ concentration as non PEMF irradiated cells which served as the controls.

### Cell culture

MSCs were extracted from human adipose tissues of 5 healthy women and 5 healthy men (age 21–36 years, body mass index 30–38) who were undergoing cosmetic surgery procedures, following the guidelines of the University of Padova’s Plastic Surgery Clinic. The adipose tissues were digested with 0.075% collagenase (type 1 A; Sigma Aldrich, Italia) in a modified Krebs-Ringer buffer [125 mM NaCl, 5 mM KCl, 1 mM Na_3_PO_4_, 1 mM MgSO_4_, 5.5 mM glucose, and 20 mM HEPES (pH 7.4)] for 60 min at 37 °C, followed by 10 min with 0.25% trypsin. Floating adipocytes were discarded, and cells from the stromal-vascular fraction were pelleted, rinsed with media, and centrifuged, after which a red cell lysis step in NH4Cl was run for 10 min at room temperature. The resulting viable cells were counted using the trypan blue exclusion assay and seeded at a density of 10^6^ cells per cm² for *in vitro* expansion in Dulbecco’s modified Eagle’s medium (DMEM, SIGMA Aldrich Italia) supplemented with 10% fetal calf serum and 1% penicillin/streptomycin. For treatment in inflammatory conditions, the cells were treated for 24 h with 0.1 mg/mL^−1^ of tumor necrosis factor-alpha (Celbio). TNF-α concentration used in the study is higher than in physiologic conditions. However, the aforementioned concentration was chosen based on the previously published papers in order to achieve effects in *in-vitro* studies^38,39^.

### DNA content

DNA content was determined using a DNeasy kit (Qiagen, Hilden, Germany) to isolate total DNA from cell cultures following the manufacturer’s protocol for tissue isolation, using overnight incubation in proteinase K (Qiagen). DNA concentration was detected by measuring the absorbance at 260 nm in a spectrophotometer. The cell number was then determined from a standard curve (microgram DNA vs. cell number) generated by DNA extraction from the counted cells. The standard curve was linear over the tested range of 5–80 µg DNA (r = 0.99).

### MTT assay

To determine the proliferation rate of cell growth on titanium disks with or without treatment, a methyl thiazolyl-tetrazolium (MTT)-based cytotoxicity assay was performed according to the method of Denizot and Lang with minor modifications^40^. The test is based on mitochondria viability, i.e., only functional mitochondria can oxidize an MTT solution, giving a typical blue-violet endproduct. After harvesting the culture medium, the cells were incubated for 3 h at 37 °C in 1 mL 0.5 mg/mL MTT solution prepared in phosphate buffered saline (PBS) solution. After removal of the MTT solution by pipette, 0.5 mL 10% dimethyl sulfoxide in isopropanol (iDMSO) was added for 30 min at 37 °C. For each sample, absorbance values at 570 nm were recorded in duplicate on 200 μL aliquots deposited in 96-well plates using a multilabel plate reader (Victor 3 Perkin Elmer, Milano, Italy). All samples were examined after 15 and 30 days of culture^40^.

### RNA extraction and first-strand cDNA synthesis

RNase-Free DNase Set (Qiagen) from implants were cultured with adipose tissue derived mesenchymal stem cells for 15 and 25 days. The RNA quality and concentration of the samples were measured using a NanoDrop^TM^ ND-1000 Spectrophotometer (Thermo Scientific). For the first-strand cDNA synthesis, 200 ng of total RNA of each sample was reverse transcribed with M-MLV Reverse Transcriptase (Invitrogen), following the manufacturer’s protocol.

### Real-time PCR

Human primers were selected for each target gene with Primer 3 software (Table 1). Real-time PCRs were carried out using the designed primers at a concentration of 300 nM and FastStart SYBR Green Master (Roche) on a Rotor-Gene 3000 (Corbett Research, Sydney, Australia). Real-time PCR was performed also according to the user’s manual for the Human mTOR signaling Profiler PCR Array (SABiosciences, Frederick, MD, USA) and using RT2 SYBR Green ROX FAST Master Mix (Qiagen). The data were analyzed using Excel-based PCR Array Data Analysis Templates (SABiosciences). The thermal cycling conditions were as follows: 15 min denaturation at 95 °C, followed by 40 cycles of 15 s denaturation at 95 °C, annealing for 30 s at 60 °C, and 20 s elongation at 72 °C. Differences in gene expression were evaluated by the 2∆∆Ct method, using MSCs cultured in the presence and absence of inflammatory cytokines and in the presence and absence of PEMFs. Values were normalized to the expression of glyceraldehyde-3-phosphate dehydrogenase (GAPDH) internal reference whose abundance did not change under our experimental conditions. Experiments were performed with 3 different cell preparations and repeated at least 3 times.

**Table 1:** List of gene related to mTOR pathway analized by RT PCR.

	Description	Gene
mTOR1 Complexes:	MTOR associated protein, LST8 homolog (S. cerevisiae)	MLST8
	Mechanistic target of rapamycin (serine/threonine kinase)	MTOR
	Regulatory associated protein of MTOR, complex 1	RPTOR
mTOR2 Complexes:	Mitogen-activated protein kinase associated protein 1	MAPKAP1
	RPTOR independent companion of MTOR, complex 2	RICTOR
mTOR Upstream Regulators negative regulation:	Eukaryotic translation initiation factor 4E binding protein 1	EIF4EBP1
	Eukaryotic translation initiation factor 4E binding protein 2	EIF4EBP2
	Protein phosphatase 2, catalytic subunit, alpha isozyme	PPP2CA
	Protein phosphatase 2, regulatory subunit B, beta	PPP2R2B
	Protein phosphatase 2 A activator, regulatory subunit 4	PPP2R4
	Tumor protein p53	TP53
	Unc-51-like kinase 1 (C. elegans)	ULK1
	Unc-51-like kinase 2 (C. elegans)	ULK2
mTOR Upstream Regulators positive regulation:	Cell division cycle 42 (GTP binding protein, 25 kDa)	CDC42
	Conserved helix-loop-helix ubiquitous kinase	CHUK
	Eukaryotic translation initiation factor 4B	EIF4B
	Eukaryotic translation initiation factor 4E	EIF4E
	Glycogen synthase kinase 3 beta	GSK3B
	Hypoxia inducible factor 1, alpha subunit (basic helix-loop-helix transcription factor)	HIF1A
	Heat shock 70 kDa protein 4	HSPA4
	Integrin-linked kinase	ILK
	Myosin IC	MYO1C
	Protein kinase C, alpha	PRKCA
	Protein kinase C, beta	PRKCB
	Protein kinase C, epsilon	PRKCE
	Protein kinase C, gamma	PRKCG
	Ras homolog gene family, member A	RHOA
	Ribosomal protein S6	RPS6
	Ribosomal protein S6 kinase, 70 kDa, polypeptide 1	RPS6KB1
	Ribosomal protein S6 kinase, 70 kDa, polypeptide 2	RPS6KB2
	Serum/glucocorticoid regulated kinase 1	SGK1
	Vascular endothelial growth factor A	VEGFA
	Vascular endothelial growth factor B	VEGFB
	Vascular endothelial growth factor C	VEGFC
mTOR Downstream Effectors negative regulation:	AKT1 substrate 1 (proline-rich)	AKT1S1
	Calcium binding protein 39	CAB39
	Calcium binding protein 39 L	CAB39L
	DNA-damage-inducible transcript 4	DDIT4
	DNA-damage-inducible transcript 4-like	DDIT4L
	DEP domain containing MTOR-interacting protein	DEPTOR
	FK506 binding protein 1 A, 12 kDa	FKBP1A
	FK506 binding protein 8, 38 kDa	FKBP8
	Insulin-like growth factor binding protein 3	IGFBP3
	Protein kinase, AMP-activated, alpha 1 catalytic subunit	PRKAA1
	Protein kinase, AMP-activated, alpha 2 catalytic subunit	PRKAA2
	Protein kinase, AMP-activated, beta 1 non-catalytic subunit	PRKAB1
	Protein kinase, AMP-activated, beta 2 non-catalytic subunit	PRKAB2
	Protein kinase, AMP-activated, gamma 1 non-catalytic subunit	PRKAG1
	Protein kinase, AMP-activated, gamma 2 non-catalytic subunit	PRKAG2
	Protein kinase, AMP-activated, gamma 3 non-catalytic subunit	PRKAG3
	Phosphatase and tensin homolog	PTEN
	Serine/threonine kinase 11	STK11
	STE20-related kinase adaptor beta	STRADB
	Tuberous sclerosis 1	TSC1
	Tuberous sclerosis 2	TSC2
	Tyrosine 3-monooxygenase/tryptophan 5-monooxygenase activation protein, theta polypeptide	YWHAQ
mTOR Downstream Effectors positive regulation:	V-akt murine thymoma viral oncogene homolog 1	AKT1
	V-akt murine thymoma viral oncogene homolog 2	AKT2
	V-akt murine thymoma viral oncogene homolog 3 (protein kinase B, gamma)	AKT3
	V-Ha-ras Harvey rat sarcoma viral oncogene homolog	HRAS
	Insulin-like growth factor 1 (somatomedin C)	IGF1
	Inhibitor of kappa light polypeptide gene enhancer in B-cells, kinase beta	IKBKB
	Insulin	INS
	Insulin receptor	INSR
	Insulin receptor substrate 1	IRS1
	Mitogen-activated protein kinase 1	MAPK1
	Mitogen-activated protein kinase 3	MAPK3
	3-phosphoinositide dependent protein kinase-1	PDPK1
	Phosphoinositide-3-kinase, class 3	PIK3C3
	Phosphoinositide-3-kinase, catalytic, alpha polypeptide	PIK3CA
	Phosphoinositide-3-kinase, catalytic, beta polypeptide	PIK3CB
	Phosphoinositide-3-kinase, catalytic, delta polypeptide	PIK3CD
	Phosphoinositide-3-kinase, catalytic, gamma polypeptide	PIK3CG
	Phospholipase D1, phosphatidylcholine-specific	PLD1
	Phospholipase D2	PLD2
	Ras homolog enriched in brain	RHEB
	Ribosomal protein S6 kinase, 90 kDa, polypeptide 1	RPS6KA1
	Ribosomal protein S6 kinase, 90 kDa, polypeptide 2	RPS6KA2
	Ribosomal protein S6 kinase, 90 kDa, polypeptide 5	RPS6KA5
	Ras-related GTP binding A	RRAGA
	Ras-related GTP binding B	RRAGB
	Ras-related GTP binding C	RRAGC
	Ras-related GTP binding D	RRAGD
	TEL2, telomere maintenance 2, homolog (S. cerevisiae)	TELO2

### Real-time PCR - mTOR

Total RNA was extracted using an RNeasy Lipid Tissue kit (Qiagen), including DNase digestion with the RNase-Free DNase. Set (Qiagen), from the mTOR signalling RT2 profiler PCR Array (gene analized are reported on Table 1). In total, 800 ng of RNA was reverse-transcribed using an RT2 First Strand kit (Qiagen). Real-time PCR was performed according to the user’s manual for the Human mTOR signalling RT2 profiler PCR Array (SABiosciences, Frederick, MD, USA) and using RT2 SYBR Green ROX FAST Master Mix (Qiagen). Thermal cycling and fluorescence detection were performed using a Rotor-Gene Q 100 (Qiagen). The data were analyzed using Excel-based PCR Array Data Analysis Templates (SABiosciences).

### Alizarin Red S staining

The extracellular mineral deposits were detected by Alizarin Red S staining. Cells were fixed in 4% paraformaldehyde (Sigma-Aldrich) in PBS for 10 min at room temperature. Cells were stained adding 40 mM freshly Alizarin Red S Solution (pH 4.2) for 10 min at room temperature with gentle shaking. Cells were washed with ddH2O, then photographed by an optical microscope. Alizarin Red S stained area were quantified from microscope images of three independent experiments using ImageJ software (NIH, Bethesda, MD, USA).

### ALP activity measurements

Alkaline phosphatase (ALP) activity was measured for up to 20 days of cell culture in order to evaluate the initial differentiation of Adipose Tissue Derived Mesenchymal Stem cells into preosteoblasts. Abcam’s alkaline phosphates kit (colorimetric) was used to detect the intracellular and extracellular ALP activities. The kit uses p-nitrophenyl phosphate (pNPP) as a phosphatase substrate, which is adsorbed at 405 nm when dephosphorylated by ALP. In accordance with the manufacturer’s protocol, the culture medium from each sample group was collected and pooled. At the same time, the cells were washed with PBS and then homogenized with ALP assay buffer (a total of 300 μL for each group) and centrifuged at 13,000 rpm for 3 min to remove insoluble material. Different volumes of samples (medium and cells) were then added into 96-well plates, bringing the total volume in each well up to 80 μL with assay buffer. In addition, 80 μL fresh medium was utilized as sample background control. Thereafter, 50 μL 5mMpNPP solution was added to each well containing test samples and background control and incubated for 60 min at 25 °C while shielding the plate from light. A standard curve of 0, 4, 6, 12, 16, and 20 nmol/well was generated from 1 mM pNPP standard solution, bringing the final volume to 120 μL with assay buffer. All reactions were then stopped by adding 20 μL of stop solution into each standard and sample reaction, except the sample background control reaction. Optical density was read at 405 nm in a microplate reader (Victor). The results were normalized by subtracting the value derived from the zero standards from all standards, samples and sample background control. The pNP standard curve was plotted to identify the pNP concentration in each sample. ALP activity of the test samples was calculated as follows:

$${\rm{ALP}}\,{\rm{activity}}\,({\rm{U}}/{\rm{ml}})={\rm{A}}/{\rm{V}}/{\rm{T}}$$

where: A is the amount of pNP generated by samples (in μmol), V is the amount of sample added in the assay well (in mL), and T is the reaction time (in minutes).

### Immunofluorescence

Cells were fixed in 4% paraformaldehyde in PBS for 10 min and then incubated in 2% bovine serum albumin (BSA, Sigma-Aldrich) in PBS for 30 min at room temperature. They were then incubated with primary antibodies in 2% BSA solution in a humidified chamber for 12 h at 4 °C. The rabbit polyclonal antihuman phalloidine antibody (Millipore Corporation, MA, USA) was the primary antibody. Immunofluorescence staining was performed using the secondary antibody DyLight 549-labeled anti-rabbit IgG (H + L) (KPL, Gaithersburg, MD, USA) diluted 1/1000 in 2% BSA for 1 h at room temperature. Nuclear staining was performed with 2 μg/mL Hoechst H33342 (Sigma-Aldrich) solution for 2 min.

### Statistical analysis

One-way analysis of variance (ANOVA) was used for data analyses. Levene’s test was used to demonstrate the equal variances of the variables. Repeated measures ANOVA with a post-hoc analysis using Bonferroni’s multiple comparison was performed. T-tests were used to determine significant differences (p < 0.05). Repeatability was calculated as the standard deviation of the difference between measurements. All testing was performed using SPSS 16.0 software (SPSS Inc., Chicago, IL, USA) (license of the University of Padua, Italy).
